# Birth Pattern Seasonality in Ethiopia: Evidence from National Demographic and Health Survey Data from 2000 to 2019

**DOI:** 10.1089/whr.2025.0024

**Published:** 2025-05-07

**Authors:** Bezawit Alemu Bezabih, Mulugeta Shegaze Shimbre, Tamirat Gezahegn Guyo, Mesfin Mamo Utaile, Manaye Yihune, Aynalem Yemane Leyew, Getahun Koira Kolbaye, Habtamu Esubalew Bezie

**Affiliations:** ^1^ School of Public Health, College of Medicine and Health Science, Arba Minch University, Arba Minch, Ethiopia.; ^2^ School of Public Health, Cheeloo College of Medicine, Shandong University, Jinan, China.; ^3^ Department of Public Health, Arba Minch College of Health Sciences, Arba Minch, Ethiopia.; ^4^ Department of Pediatric and Child Health, School of Medicine, College of Medicine and Health Sciences, Arba Minch University, Arba Minch, Ethiopia.; ^5^ School of Medicine, College of Medicine and Health Sciences, Arba Minch University, Arba Minch, Ethiopia.

**Keywords:** Birth, Seasonality pattern, Secondary data, Ethiopia, Demographic Health Survey

## Abstract

**Background:**

The periodicity of births and the factors that influence them have not been thoroughly investigated in Ethiopia. Hence, this study aimed to assess birth seasonality patterns in Ethiopia using data collected from Demographic and Health Surveys over the past two decades (2000–2019).

**Methods:**

A descriptive cross-sectional study was employed to record Ethiopian birth seasonality in greater detail than has previously been accomplished. The Demographic and Health Survey birth data were used to systematically document, evaluate, and compare the birth seasonality in Ethiopia.

**Results:**

Nationally, there was an early peak in the year, followed by a gradual decline. Regarding regional variation, in Afar and Somalia, the birth patterns show high variation, and Tigray, Amhara, Addis Ababa, and South Nation Nationality and Peoples Region exhibit relatively low variation in birth patterns, respectively.

**Conclusions:**

The birth pattern is not uniform and varies seasonally and with different locations as well as maternal demographics. This finding could assist in the prediction of seasonal birth rates, guide contraception campaigns, distribute vaccinations strategically, and design a proactive measure against childhood diseases using mathematical modeling.

## Introduction

Human reproduction occurs throughout the year, but there are seasonal fluctuations in birth rates, with some months experiencing higher rates than others.^1^ This pattern is common in human communities and can lead to variations in monthly birth rates with different peaks and ranges. Seasonal patterns of births reflect seasonal variations in conception, driven by fluctuations in sexual intercourse frequency and fecundity.^2,3^ These seasonal fluctuations in birth patterns can play a role in infectious disease dynamics by increasing the number of susceptible individuals entering communities and, thus, the likelihood of transmission.^4^

Previous studies have documented seasonal and yearly trends in birth outcomes for various populations in different geographic locations.^2,5–9^ The seasonality of births has been studied extensively in North America, Europe, and East Asia, but less so in African contexts. Although some exceptions exist, most African countries exhibit seasonal birth patterns, with high peaks observed in some areas. Factors such as a mother’s education, religion, and location can impact birth seasonality, with stronger seasonality observed in Central and West Africa and weaker seasonality in Eastern and Southern Africa.^1^ In Ethiopia, the median birth interval between births is 35.8 months, with a longer delay in urban regions than in rural (38.6 months vs. 35.1 months). The median age at first birth among women aged 25–49 is 18.7 years.^10^ The impact of contraceptives on fertility prevention was found to be 28.5% and 30.7% lower than the biological threshold in 2011 and 2016, respectively. Postpartum infertility reduced overall fertility by 34.7% in 2011 and 35% in 2016. The index of fetal wastage inhibited just 9.2% of total births in both survey years.^11^

Various environmental and behavioral processes have been suggested as reasons for seasonal differences in sexual intercourse frequency and energy expenditure with fecundity. In addition, miscarriage following infectious diseases can impact fertility and fetal loss.^5,9,12^ Delaying initial deliveries and increasing the gap between pregnancies have played a crucial role in lowering fertility rates in various countries and improving maternal and child health outcomes. Early childbearing is associated with an increased risk of problems during pregnancy and childbirth, as well as higher rates of infant mortality.^10^

Generally, seasonal fluctuations in birth patterns are common in human communities and can have major implications for infectious disease dynamics. A variety of factors influence the number of children a woman carries, and delayed initial deliveries and increased birth intervals have been shown to improve maternal and child health outcomes. Improving access to contraceptives can also help to lower fertility rates and improve maternal and child health outcomes. Recent studies have also examined the relationship between birth month and later life outcomes, such as infant mortality, life expectancy, female fecundity, educational attainment, and salary, piquing the interest of epidemiologists and social scientists in birth seasonality.^13,14^ However, many of these studies assume that the month or quarter of birth is distributed randomly across the population, whereas changes in women’s seasonal fertility rates may result in differences in newborns even before conception. Variations in individual qualities or family background may, therefore, underlie seasonality in outcomes.^15^

Moreover, the seasonality of birth can also impact other systems, including infectious disease dynamics. Studies have shown that the amplitude of seasonal birth trends can influence the timing and scale of pediatric illness outbreaks, such as measles,^16^ leading to improved control strategies in sub-Saharan African countries such as Ethiopia, where no studies on birth seasonality have been identified thus far. To address this problem, this study aims to examine birth pattern seasonality to describe the distribution of birth patterns in Ethiopia over the past two decades and across various maternal demographic characteristics. By understanding birth seasonality in Ethiopia, this study can contribute to a broader understanding of birth seasonality in African contexts and its impact on infectious disease dynamics, maternal and child health outcomes, and other social and economic factors.

## Materials and Methods

### Study design and period

A descriptive analytical approach was employed to comprehensively document the birth seasonality in Ethiopia. The study utilized data collected from five rounds of the Ethiopian Demographic and Health Survey (EDHS) conducted in 2000, 2005, 2011, 2016, and 2019. The Demographic and Health Surveys (DHS) is an open-access database that provides comprehensive information on various topics with national and regional representation in the developing world. The DHS employed a two-stage stratified probability sampling approach using an up-to-date census as the sample frame. Clusters of dwellings were selected, and the DHS provided the necessary information, such as stratum, primary sampling unit, and sample weights, to account for survey design and participation. The primary focus of the analysis was the Ethiopian 2016 DHS dataset, whereas data from the other survey years (2000, 2005, 2011, and 2019) were used for result comparison and to assess consistency across different data collection periods.

### Study area and population

The study was conducted in Ethiopia. Ethiopia is a landlocked country located in the Horn of Africa. Ethiopia is the second-most populous country in Africa after Nigeria. Ethiopia’s climate is diverse, ranging from equatorial rainforest in the south and southwest to desert regions in the northeast, east, and southeast. These diverse climatic conditions within Ethiopia contribute to the country’s sociocultural and agricultural diversity.^17^ The study adopted a cross-sectional study design, utilizing birth history records obtained from women aged 15–49 to analyze the birth months of their children. The DHS birth history data contained detailed information on the month and year of birth for each delivery made by women aged 15–49 who participated in the survey, enabling a comprehensive analysis of birth history. Individual women’s datasets also contained sociodemographic information about the mothers.

### Sampling method

The sampling process for the 2016 EDHS was based on the census enumeration areas (EAs) established for the 2016 Ethiopian Population and Housing Census (EPHC) conducted by the Central Statistical Agency (CSA). The census frame consisted of a comprehensive list of EAs created specifically for the 2016 EPHC, with an average number of households per EA. Each EA in the sample frame provided details regarding its location, type of residence (urban or rural), and the expected number of residential households. Ethiopia was divided into nine administrative regions and two administrative cities, and the sampling design for the 2016 EDHS aimed to generate estimates for the entire country, as well as for urban and rural areas, each region, and the two administrative cities. The selection of the sample for the 2016 EDHS involved a two-phase process. The regions were first stratified into distinct urban and rural areas, creating separate sample strata. Within each stratum, EAs were chosen in two phases using implicit stratification and proportional allocation. The sampling frame was organized based on various administrative units, and the first stage of sampling involved selecting EAs with a probability proportional to their size, as determined by the 2016 EPHC frame.

During the data collection phase, house listing activities were carried out, and the collected household lists served as the sample frame for selecting households. In some cases, certain EAs chosen for the 2016 EDHS contained more than 300 families, prompting the division of these EAs to simplify the house listing process.^18^

### Secondary data acquisition

Upon accessing the DHS Program website (https://www.dhsprogram.com), a thorough exploration was conducted to identify and select the Ethiopia-specific survey dataset of interest. The accompanying documentation, encompassing questionnaires, survey reports, and data dictionaries, was carefully reviewed to comprehend the variables and data collection methodologies specifically applicable to Ethiopia. The data files, formatted in Stata, were then downloaded from the website. Subsequently, the files were extracted and imported into the Stata software for further analysis and visualization.

### Study variables

The dependent variable was birth seasonality pattern, and the independent variables included maternal age, maternal educational status, maternal religion, maternal socioeconomic status, area of living, marital status, and occupation.

### Operational definition

**Birth seasonality patterns:** The birth months collected from mothers in a given year are aggregated to evaluate the proportion of children born in a given month.

**Fecundity:** The ability to conceive or produce an abundance of offspring.

**Unimodal birth seasonality peak:** A birth pattern seasonality peak at one time in a year.

**Bimodal birth seasonality peak:** A birth pattern seasonality peak occurs two times in a year.

**Uniform birth pattern:** A birth pattern with minimal to no difference among the proportion of children born in different months of the year.

### Data processing and analysis

The obtained datasets from the Ethiopian DHS were processed and analyzed using STATA/SE 17.0. The primary focus of the analysis was the Ethiopian DHS 2016 dataset, which contained valid data on basic demographic information of women aged 15–49 and birth month histories. To address the nonproportional allocation of the sample to different regions and potential differences in response rates, a sampling weight was applied. To analyze the descriptive demographic information of the mothers, individual women’s datasets were used. For the analysis of birth month histories, the births dataset was utilized, treating the number of children ever delivered as the unit of entry. This approach accounted for the possibility of multiple children per woman. To ensure accurate analysis, variables were adjusted to account for the complex survey design and appropriate survey weights. The births dataset included a comprehensive history of children ever born to the mothers included in the study, including the birth month and year. Descriptive statistics, data visualization, and summary measures were utilized to assess the distribution, range, and patterns within the variables. This exploratory analysis provided valuable insights into the overall characteristics and quality of the data. It is important to note that the data were collected in the Ethiopian calendar, specifically in months and years. Therefore, the analysis was conducted using the Ethiopian calendar and was not converted into the Gregorian calendar.

### Ethical approval

Ethical approval was obtained from the Ethical Review Board (institutional review board) of the College of Medicine and Health Science at Arba Minch University. To obtain the necessary datasets from the DHS, we submitted a formal request and obtained a permission letter. It is important to note that all participants in the original DHS study provided informed consent before their involvement, ensuring that their rights and privacy were respected.

## Results

### Maternal demographic characteristics

A total of 15,683 mothers were included in the study. The age distribution revealed that a noteworthy proportion of mothers (21.56%) were under the age of 19, whereas the largest group fell within the 20–29 age range (36.47%). A total of 34.70% of the mothers belonged to the poor socioeconomic category, and the majority of mothers identified as Orthodox Christians (43.27%). Nearly half of the mothers (47.81%) reported no formal education, and the largest proportion of mothers (36.35%) resided in the Oromia region. Geographically, the majority of mothers (77.84%) lived in rural areas. Occupationally, the largest group of mothers (49.86%) was not engaged in any formal employment, and the majority (63.85%) were married (Table 1).

**Table 1.** Maternal Sociodemographic Characteristics of the Mothers in Ethiopia, Ethiopian Demographic and Health Survey 2016

Variables	Categories	Weighted frequency (*n* = 15,683)	Weight relative frequency (%)
Age in years	<19	3,380.0	21.6
	20–29	5,719.0	36.5
	30–39	4,275.0	27.3
	>40	2,309.0	14.7
Socioeconomic status	Poor	5,442.0	34.7
	Middle	6,077.8	38.8
	Rich	4,163.2	26.6
Religion	Orthodox	6,786.2	43.3
	Protestant	3,674.1	23.4
	Muslim	4,892.7	31.2
	Others^a^	330.0	2.1
Region	Tigray	1,129.0	7.2
	Afar	128.2	0.8
	Amhara	3,714.1	23.7
	Oromia	5,700.9	36.4
	Somali	459.5	2.9
	Benishangul	160.4	1.0
	SNNPR	3,288.0	21.0
	Gambelia	43.7	0.3
	Harar	38.5	0.3
	Addis Ababa	930.3	5.9
	Dire Dawa	90.4	0.6
Location	Urban	3,476.0	22.2
	Rural	12,207.0	77.8
Educational status	No formal education	7,497.9	47.8
	Primary	5,490.4	35.0
	Secondary and above	2,694.7	17.2
Occupation	Not working	7819.4	49.9
	Government employee	3420.7	21.8
	Farmer	3263.3	20.8
	Others^b^	1179.6	7.5
Marital status	Single	4,036.4	25.7
	Married	10,014.3	63.9
	Other^c^	1,632.2	10.4

^a^ Jehovah’s Witness, cultural religion, and pagan.

^b^ Daily laborer and nongovernmental organizations.

^c^ Divorced and widowed.

SNNPR, South Nation Nationality and Peoples Region.

### Birth seasonality pattern

#### Nationally

The highest percentage of births occurred in September, accounting for 10.44% of the total. This was followed closely by November with 10.16% and January with 10.14%. Conversely, the months of July (6.17%) and August (5.35%) had the lowest percentages of births. Collectively, these findings suggest a concentration of births in the earlier months of the year, with September, October, November, and December accounting for a major proportion of the cumulative births. It is worth noting that the distribution appears relatively balanced, with most months falling within the range of 7%–10% of the total births. This suggests a relatively even distribution of births throughout the year within the studied population (Table 2).

**Table 2.** National Birth Seasonality Pattern in Ethiopia, Ethiopian Demographic and Health Survey 2016

Birth month Ethiopian calendar^a^	Birth month Gregorian calendar	Weighted relative frequency (%)	95% CI	Weighted cumulative relative (%)
Meskerem	September	10.4	10.0, 11.0	10.4
Tikimit	October	9.3	8.92, 9.8	19.8
Hidar	November	10.2	9.7, 10.6	29.9
Tahisas	December	10.1	9.5, 10.6	40.0
Tir	January	10.1	9.7, 10.6	50.1
Yekatit	February	7.6	7.2, 8.1	57.8
Megabit	March	8.1	7.6, 8.5	65.8
Miyazia	April	8.5	8.1, 9.0	74.3
Ginbot	May	7.1	6.7, 7.6	81.5
Sene	June	7.0	6.7, 7.5	88.5
Hamle	July	6.2	5.8, 6.6	94.7
Nehase	August	5.4	5.1, 5.7	100.0

^a^ The Ethiopian calendar has 13 months; the extra month dates were redistributed across the other months to align with the corresponding number of dates in the Gregorian calendar.

CI, confidence interval.

#### Birth seasonality patterns by regions

**FIG. 1.**
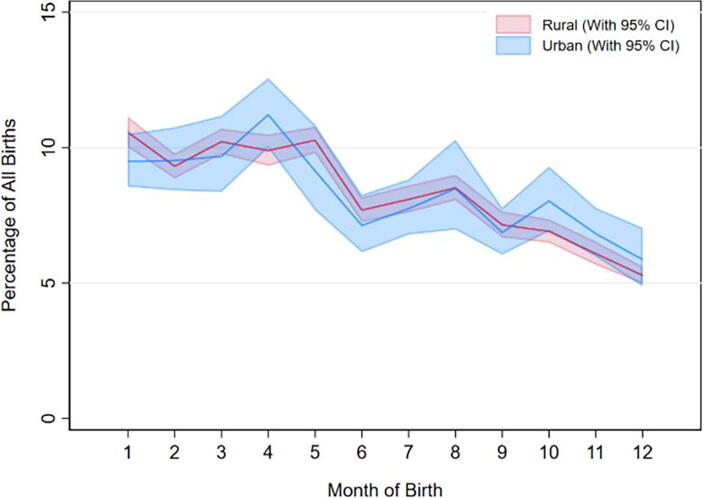
Birth seasonality pattern by area of living (urban/rural) in Ethiopia, EDHS 2000 to 2019. EDHS, Ethiopian Demographic and Health Survey.

When comparing the birth percentages in different regions of Ethiopia to an assumed ideal proportion of uniform births (8.33% for each month), certain regions demonstrate closer proximity to this ideal, whereas others exhibit higher variation. In regions such as Afar and Somalia, the birth rates show high variation, with rates ranging widely from 2.15 to 14.5 births per month and 2.59 to 14.15 births per month, respectively. On the contrary, Tigray, Amhara, Addis Ababa, and South Nation Nationality and Peoples Region (SNNPR) show relatively low variation in birth rates, with ranges of 6.39–9.82, 6.41–10.01, 7.12–10.42, and 5.84–11.44 births per month, respectively (Table 3) (Fig. 1).

**Table 3.** Weighted Birth Seasonality Pattern by Region in Ethiopia, Ethiopian Demographic and Health Survey 2016

Birth month^a^	Tigray	Afar	Amhara	Oromia	Somali	Benishangul	SNNPR	Gambelia	Harari	Addis Ababa	Dire Dewa
September	8.2	13.1	8.6	10.9	14.2	10.3	11.4	10.6	11.4	9.1	11.2
October	8.1	12.3	8.7	10.3	12.2	9.1	8.3	8.5	10.1	7.3	9.6
November	8.4	14.5	9.4	10.7	11.8	10.6	10.1	10.2	11.2	8.4	10.0
December	8.0	13.9	9.4	10.7	11.2	9.6	9.7	10.3	10.2	9.7	11.1
January	9.1	12.0	10.0	10.1	11.6	11.6	10.4	11.3	11.0	8.2	10.9
February	8.1	10.7	7.1	7.5	9.0	10.4	7.5	10.0	9.0	10.4	8.9
March	9.1	7.3	8.45	7.9	8.9	8.2	7.4	7.4	7.0	8.8	7.1
April	8.9	4.5	9.31	8.2	7.1	7.2	8.6	9.48	7.5	9.1	6.5
May	9.8	3.0	8.1	6.5	5.2	6.5	7.1	7.3	6.4	7.1	7.4
June	9.2	3.4	7.7	6.8	3.2	7.0	7.0	5.6	5.9	7.6	6.1
July	6.7	3.3	6.8	5.9	3.1	5.1	6.7	4.6	6.1	6.3	6.6
August	6.4	2.2	6.4	4.6	2.6	4.4	5.8	4.8	4.4	8.0	4.7

^a^ The Ethiopian calendar has 13 months; the extra month dates were redistributed across the other months to align with the corresponding number of dates in the Gregorian calendar.

#### Estimated conception dates in the regions

There was a universal increase in conception around December in all regions. In general observations, it is noteworthy that December, January, and February tend to have higher conception percentages across several regions. This suggests a possible seasonal influence on conception. The region of Afar and Somalia consistently exhibits a lower percentage of conceptions during September, October, and November when compared with other months. In contrast, the regions of Tigray, Amhara, SNNPR, and Addis Ababa do not exhibit any major peaks or troughs in conception percentages. Instead, the data show relatively consistent percentages of conceptions across the months, indicating a more stable pattern of conception in these regions (Table 4) (Fig. 1).

**Table 4.** Estimated Conception Date in the Regions in Ethiopia, Ethiopian Demographic and Health Survey 2016

Month	Tigray	Afar	Amhara	Oromia	Somali	Benishangul	SNNPR	Gambelia	Harari	Addis Ababa	Dire Dewa
September	9.2	3.4	7.7	6.8	3.2	7.0	7.0	5.6	5.9	7.6	6.1
October	6.7	3.3	6.8	5.9	3.1	5.1	6.7	4.6	6.1	6.3	6.6
November	6.4	2.2	6.4	4.6	2.6	4.4	5.8	4.8	4.4	8.0	4.7
December	8.2	13.1	8.6	10.9	14.2	10.3	11.4	10.6	11.4	9.1	11.2
January	8.1	12.3	8.7	10.3	12.2	9.1	8.3	8.5	10.1	7.3	9.6
February	8.4	14.5	9.4	10.7	11.8	10.6	10.1	10.2	11.2	8.4	10.0
March	8.0	13.9	9.4	10.7	11.2	9.6	9.7	10.3	10.2	9.7	11.1
April	9.1	12.0	10.0	10.1	11.6	11.6	10.4	11.3	10.95	8.17	10.9
May	8.1	10.7	7.1	7.5	9.0	10.4	7.5	10.0	9.0	10.4	8.9
June	9.1	7.3	8.5	7.9	8.9	8.2	7.4	7.4	7.0	8.8	7.1
July	8.9	4.5	9.3	8.2	7.1	7.2	8.6	9.4	7.5	9.1	6.5
August	9.8	3.0	8.1	6.5	5.2	6.5	7.1	7.3	6.4	7.1	7.4

#### Birth seasonality pattern by area of living (urban or rural)

The proportion of births in urban areas varies across the months, ranging from 5.87% (August) to 11.22% (December), with corresponding confidence intervals of 4.88%–7.04% and 9.99%–12.57%, respectively. In rural areas, the proportion of births ranges from 5.28% (August) to 10.57% (September), with confidence intervals of 4.97%–5.61% and 10.0%–1.14%, respectively. It is important to note that the confidence intervals provide a measure of uncertainty around the estimated proportions, and overlapping intervals suggest that the observed differences in proportions may not be statistically significant (Fig. 1).

#### Birth seasonality pattern by maternal education

**FIG. 2.**
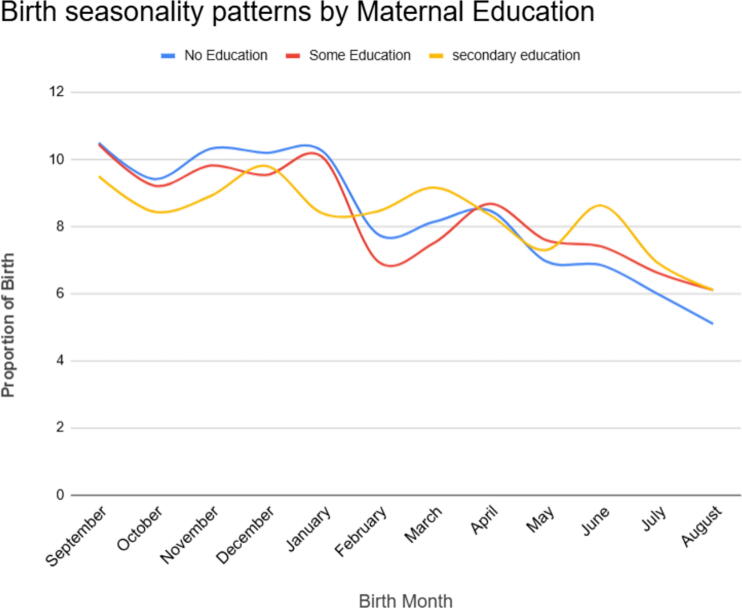
Birth seasonality pattern based on maternal educational status in Ethiopia, EDHS 2000 to 2019.

The findings indicate some variations in birth seasonality patterns based on maternal education levels. In general, higher levels of maternal education appear to be associated with slightly lower birth percentage variations. For example, in September, birth percentages decreased with higher levels of education, with 10.5% for “no education,” 10.45% for “some education,” and 9.5% for “secondary education.” Higher levels of maternal education were associated with slightly lower birth percentage variations. However, the differences between education levels are relatively small (Fig. 2).

#### Birth seasonality pattern based on socioeconomic status

**FIG. 3.**
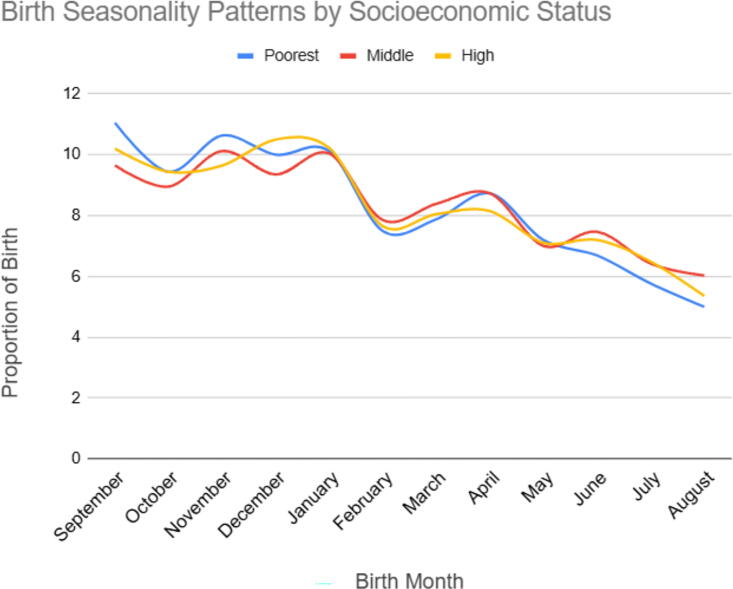
Birth seasonality pattern based on socioeconomic status in Ethiopia, EDHS 2000 to 2019.

The birth percentages for different months are categorized based on the socioeconomic status of the mother. The findings indicate some variations in birth seasonality patterns based on socioeconomic status. In general, there are slight differences in birth percentages across different socioeconomic groups for certain months. For example, in September, the highest birth percentage was observed among the poorest group (11.05%), followed by the richer group (10.2%), and the middle group (9.65%). However, it is important to note that the differences between socioeconomic groups are relatively small (Fig. 3).

#### Birth seasonality pattern based on maternal age

In general, there are slight differences in birth percentages across different age groups for certain months. For example, in September, the highest birth percentage is observed among mothers aged 30–34 (11.48%), followed by mothers aged 15–24 (11.18%), and mothers aged 35–39 (10.24%) (Table 5).

**Table 5.** Birth Seasonality Pattern by Maternal Age in Ethiopia, Ethiopian Demographic and Health Survey 2016

Birth month	Maternal age (weighted)					
	15–24	25–29	30–34	35–39	40–44	45–49
September	11.2	10.4	11.5	10.2	10.0	9.6
October	9.2	9.3	9.0	8.9	10.2	9.5
November	9.8	9.4	10.2	10.7	10.4	9.9
December	9.4	10.1	9.8	10.6	10.0	9.9
January	9.5	10.1	10.7	10.0	10.3	9.8
February	7.4	7.3	7.8	7.9	7.4	7.8
March	7.6	8.5	7.7	8.0	8.0	8.4
April	7.6	9.0	8.8	8.6	8.0	8.5
May	7.5	7.4	6.9	6.8	7.3	7.3
June	7.6	7.6	6.9	7.0	6.6	7.2
July	6.4	5.8	5.8	6.5	6.4	6.2
August	6.9	5.2	5.0	4.8	5.6	5.9

#### Birth seasonality patter marital status

**FIG. 4.**
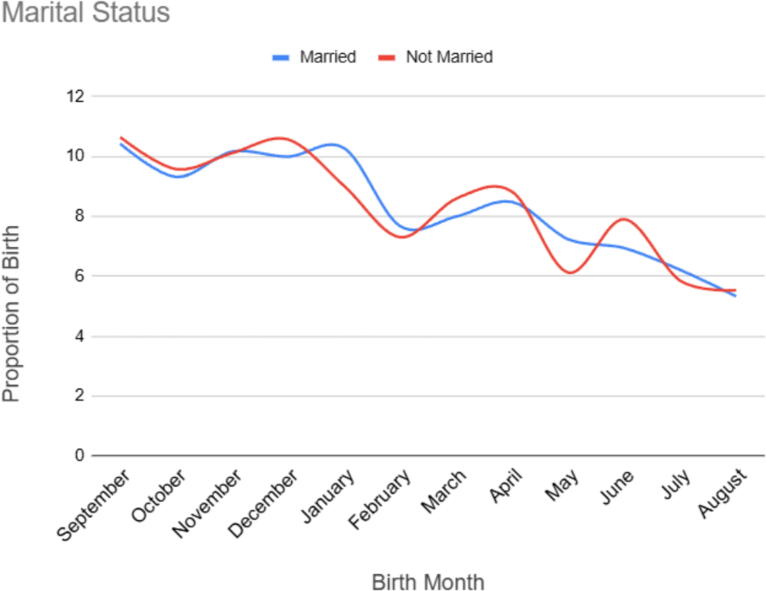
Birth seasonality pattern by marital status in Ethiopia, EDHS 2000 to 2019.

The findings indicate some variations in birth seasonality patterns based on marital status. In general, there are slight differences in birth percentages across different marital status groups for certain months. For example, in September, the highest birth percentage was observed among mothers who were not married (10.64%) (Fig. 4).

#### Birth seasonality pattern by different EDHS years

**FIG. 5.**
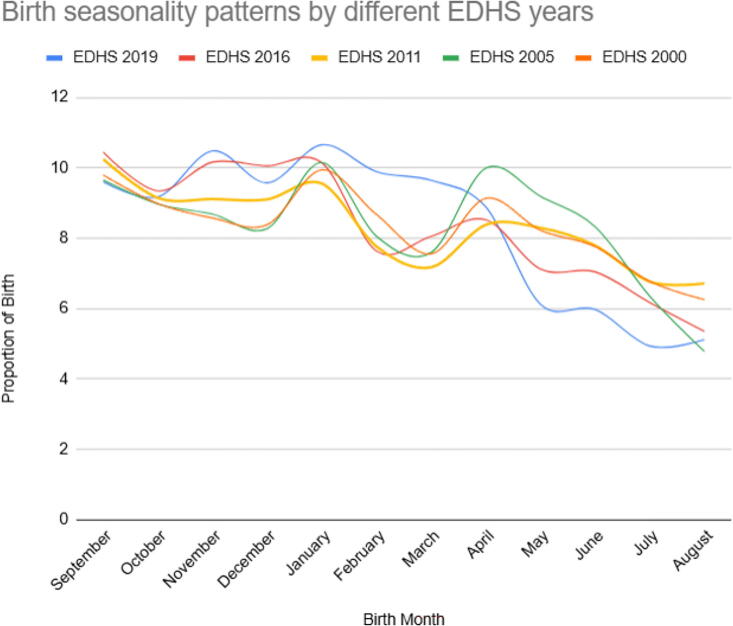
Birth seasonality pattern at different years of EDHS in Ethiopia, EDHS 2000 to 2019.

Analyzing the birth peaks observed in different years of the DHS conducted between 2000 and 2019 reveals both similarities and differences in the seasonal patterns of births. One similarity across the survey years was the consistent peak in births during November, which tends to occur in November or December. In terms of differences, there are variations in the magnitude of peaks across different survey years. For example, the birth peak in November appears to be more pronounced in the 2019 survey compared with earlier years, indicating a potential increase in conceptions during that period in recent times. In addition, the month of February shows a distinctive difference between the 2019 survey and the earlier years. In 2019, there was a notable decrease in births during February compared with the other years, which may indicate a shift in the timing of conceptions during that month. Furthermore, there are variations in the birth peaks observed in other months across the different survey years. For instance, the month of March shows a decreasing trend in births over the years, whereas June displays a fluctuating pattern. Overall, although there are some consistent patterns in birth peaks, such as the prominence of November and December, there are also variations and differences in the seasonal patterns of births across the different survey years (Fig. 5).

## Discussion

The objective of this study was to assess the seasonality of birth patterns in Ethiopia over the past two decades. By analyzing data from the EDHS and focusing on specific objectives, including describing the seasonality of births, assessing birth peaks by regions, examining variations across different survey years, and identifying determinants of birth seasonality patterns, valuable insights were gained.

The 2016 EDHS revealed a higher birth frequency in the first 4 months of the Ethiopian calendar, accounting for around 40% of all births. This trend is consistent with previous DHS analyses conducted in 2000, 2005, 2011, and 2019. In addition, the analysis of the 2016 DHS survey data identified distinct patterns in birth seasonality. Conversely, the months of July and August had the lowest percentages of births, suggesting a relatively lower birth rate during that period. These findings provide valuable insights into the seasonal variations in birth patterns. The peak of births in September was observed in other studies as well in the developed world. A study in Europe and America reveals two global patterns of birth seasonality: European exceeds during spring and summer, peaking in September, and America exhibits a trough in April–May and peaks in September.^19^ The high birth patterns seen in September in this study could have a similar peak as observed in both European and American peaks, and more investigation could reveal the similar patterns observed in these regions.

Research indicates that the seasonality of births is associated with changes in local temperature. Regions with extreme temperatures often experience two peaks in births each year. For example, data from the early 1900s showed two distinct birth peaks per year in West Greenland and Eastern Europe.^20^ The wide variety of temperatures could be one reason for the high variety of birth patterns across the regions in Ethiopia. Some regions, such as SNNPR, showed relatively less diverse patterns, whereas Afar and Somali had high peaks earlier in the year.

The findings indicate a notable peak in births during September, which suggests that conceptions likely took place around December. This observation aligns with previous research that has proposed a potential association between the return of farmers from seasonal labor migration and an increased likelihood of conceptions during that time. Furthermore, this pattern helps us to elucidate the lower occurrence of births later in the year when farmers typically migrate for labor purposes. However, it is crucial to acknowledge the potential presence of bias in the data, which could impact the observed decline in births during the latter part of the year.^21,22^

The analysis of Ethiopian birth patterns reveals seasonality in reproductive patterns, with higher births in specific months such as September, October, November, and December. This understanding aids in planning health care resources and implementing public health interventions, considering local factors. For instance, Tigray and Afar exhibit distinct peaks in births during November and January, whereas Oromia, Somali, and Benishangul experience peaks during September. This knowledge can inform targeted interventions and strategies to address region-specific reproductive health challenges and improve maternal and child health outcomes.

Identifying conception-boosting months can enhance family planning services, enabling informed reproductive health choices and preventing unintended pregnancies. Seasonal factors and socioeconomic conditions can influence birth patterns, impacting fertility rates. Understanding these factors is crucial for developing effective public health interventions that address the needs of different subpopulations.^23,24^

The analysis of birth seasonality patterns across different survey years provides valuable insights into the stability or changes observed in birth peaks over time, as well as the potential reasons behind these variations. Examining the data, it is evident that there are both similarities and differences in birth seasonality patterns across the survey years.

One similarity observed is the consistent peak in births from September to January across all the survey years. This suggests a relatively stable pattern of increased conceptions during a specific period, which may be influenced by various factors such as cultural practices, holidays, or seasonal changes in social behaviors. Several studies propose that the seasonality of births may be influenced by various factors, including the frequency and timing of sexual intercourse, which can be influenced by socioeconomic status, preferences for wedding timing, cultural practices, and holiday periods. Alternatively, factors that can affect fertility or interrupt pregnancies, such as environmental elements such as temperature variability, as well as patterns of disease transmission, could also contribute to birth seasonality.^6,25–27^ However, there are also notable differences in birth peaks between survey years. For instance, the month of September shows a decline in the proportion of births over time, with a notable decrease observed in the most recent survey year (EDHS 2019). This decline may be attributed to sociodemographic changes, health care interventions, or policy shifts that have influenced reproductive behaviors, access to contraception, or family planning practices.^18,21^

To interpret the public health importance of these findings, it is crucial to consider the implications of birth seasonality patterns on health care planning and resource allocation. Stable or changing birth peaks can have major implications for maternal and child health services. Health facilities and health care providers need to anticipate and prepare for increased demand during peak birth months to ensure adequate staffing, infrastructure, and supplies. Moreover, understanding the reasons behind these variations can inform targeted interventions and policies to address specific challenges and promote optimal birth timing and spacing.

In conclusion, the analysis of birth seasonality patterns across different survey years provides insights into the stability or changes observed in birth peaks over time. Although certain patterns remain consistent, variations in birth peaks indicate the influence of sociodemographic changes, health care interventions, and policy shifts. Understanding these patterns and their implications is crucial for effective health care planning, resource allocation, and the design of targeted interventions to improve maternal and child health outcomes. Regions with Health and Demographic Surveillance Systems can conduct a more detailed study of birth seasonality, integrating data from immunization programs to understand seasonal variations in vaccine uptake and demand fluctuations. The inclusion of a large sample size enhances the generalizability of the findings to the overall population of Ethiopia. Longitudinal data were used to identify patterns across multiple survey years, including EDHS data from 2000, 2005, 2011, 2016, and 2019. This longitudinal approach allows for the examination of trends and changes in birth seasonality over time, providing insights into temporal patterns and potential shifts in reproductive behaviors. Standardized data collection from EDHS follows rigorous methodologies and standardized data collection procedures, ensuring the accuracy and validity of the results. Despite the strength, the study has some limitations. First, the study relies on secondary data from the EDHS. These survey data are subject to inherent limitations such as recall bias, which may affect the accuracy of reported birth months. Second, the study focuses on birth seasonality patterns without exploring the underlying factors driving these patterns, such as cultural practices, socioeconomic factors, or environmental influences. Further research is needed to explore these factors in more detail.

## Conclusions

This study has provided a comprehensive analysis of the characteristics of the maternal population and their influence on birth seasonality patterns in Ethiopia. The study revealed a considerable proportion of teenage mothers and emphasized the need for targeted interventions and reproductive health education to support this vulnerable group. In addition, the prominence of mothers in their 20s highlights the importance of tailored reproductive health services for women in this age range. These insights can guide the development of effective interventions and policies that promote reproductive health, improve maternal and child health outcomes, and address the unique needs of different subgroups within the maternal population.

## Abbreviations Used

CSA: Central Statistical Agency
DHS: Demographic and Health Surveys
EAs: Enumeration Areas
EDHS: Ethiopian Demographic and Health Survey
EPHC: Ethiopian Population and Housing Census
SNNPR: Southern Nation and Nationality Peoples Region
